# The Role of Gut-Derived Lipopolysaccharides and the Intestinal Barrier in Fatty Liver Diseases

**DOI:** 10.1007/s11605-021-05188-7

**Published:** 2021-11-03

**Authors:** Lingxuan An, Ulrich Wirth, Dominik Koch, Malte Schirren, Moritz Drefs, Dionysios Koliogiannis, Hanno Nieß, Joachim Andrassy, Markus Guba, Alexandr V. Bazhin, Jens Werner, Florian Kühn

**Affiliations:** Department of General, Visceral and Transplant Surgery, University Hospital of LMU Munich, Marchionini Str. 15, Munich, Germany

**Keywords:** NAFLD, ALD, LPS, Gut barrier, Microbiome

## Abstract

**Background:**

Hepatosteatosis is the earliest stage in the pathogenesis of nonalcoholic fatty (NAFLD) and alcoholic liver disease (ALD). As NAFLD is affecting 10–24% of the general population and approximately 70% of obese patients, it carries a large economic burden and is becoming a major reason for liver transplantation worldwide. ALD is a major cause of morbidity and mortality causing 50% of liver cirrhosis and 10% of liver cancer related death. Increasing evidence has accumulated that gut-derived factors play a crucial role in the development and progression of chronic liver diseases.

**Methods:**

A selective literature search was conducted in Medline and PubMed, using the terms “nonalcoholic fatty liver disease,” “alcoholic liver disease,” “lipopolysaccharide,” “gut barrier,” and “microbiome.”

**Results:**

Gut dysbiosis and gut barrier dysfunction both contribute to chronic liver disease by abnormal regulation of the gut-liver axis. Thereby, gut-derived lipopolysaccharides (LPS) are a key factor in inducing the inflammatory response of liver tissue. The review further underlines that endotoxemia is observed in both NAFLD and ALD patients. LPS plays an important role in conducting liver damage through the LPS-TLR4 signaling pathway. Treatments targeting the gut microbiome and the gut barrier such as fecal microbiota transplantation (FMT), probiotics, prebiotics, synbiotics, and intestinal alkaline phosphatase (IAP) represent potential treatment modalities for NAFLD and ALD.

**Conclusions:**

The gut-liver axis plays an important role in the development of liver disease. Treatments targeting the gut microbiome and the gut barrier have shown beneficial effects in attenuating liver inflammation and need to be further investigated.

## Introduction

Nonalcoholic fatty liver disease (NAFLD) is a pathologic condition defined by the deposition of triglycerides in the liver at greater than 5% of the total liver weight in the absence of excessive alcohol consumption.^1^ The term NAFLD encompasses a spectrum of pathologic conditions ranging from simple steatosis to nonalcoholic steatohepatitis (NASH), a progressive form of fatty liver disease that may lead to fibrosis, cirrhosis, hepatocellular carcinoma, and death.^2^ NAFLD is now a burgeoning health burden, and it is estimated that the prevalence of NAFLD in the general population is 24% (20–29%).^3^ Around 20–30% of NAFLD patients develop NASH, with only some of them further evolving to fibrosis and cirrhosis.^4^ NAFLD carries a large economic burden, contributes to a decreased quality of life, and is becoming a major reason for liver transplantation worldwide.^5^

Alcoholic liver disease (ALD) is a major cause of morbidity and mortality among people who abuse alcohol.^6^ Similar to NAFLD, the spectrum of ALD ranges from simple steatosis to alcoholic steatohepatitis (ASH), fibrosis, cirrhosis, and ultimately hepatocarcinoma.^7^ Alcohol causes 50% of liver cirrhosis and 10% of liver cancer related death.^8^ Besides the direct effects of alcohol on liver injury, gut microbiota plays an important role in liver damage. Alcohol intake can lead to changes in gut microbiota composition, even before the onset of liver disease.^9^

Lipopolysaccharide (LPS), also known as endotoxin, is a component of the outer cell wall of Gram-negative bacteria.^10^ In humans, the gut microbiota is the major source of LPS.^11^ LPS is generally known for its role in the induction of sepsis, septic shock, and multiple organ failure.^12^ Recently, LPS is found to be related to several other diseases, especially to metabolic disorders such as type 2 diabetes mellitus and atherosclerosis.^13,14^ Furthermore, the role of LPS in neurologic and mental disorders like Alzheimer’s disease and autism has been well described,^15,16^ linking gut microbiota to the homeostasis of the entire body.

The connection between gut microbiota and chronic liver disease has been first noticed in the 1980s when NASH was encountered as a common complication of jejunoileal bypass surgery for morbid obesity that could be reversed by treatment with metronidazole.^17^ Bacterial overgrowth in the blind loop has been considered to be responsible for liver damage. Today, the human intestinal microbiota has emerged as an important mediator of the development and progression of chronic liver diseases.

This manuscript briefly reviews the role of gut-derived LPS in the development and progression of NAFLD and ALD. Gut barrier dysfunction and dysbiosis are highlighted as they are the two major mechanisms of endotoxemia as well as potential therapeutic targets.

## The Intestinal Barrier and LPS Translocation

The intestinal barrier serves as a physical and functional barrier deterring translocation of potentially harmful luminal antigens into circulation. The four layers of the intestinal barrier include luminal intestinal alkaline phosphatase (IAP) released from the intestinal epithelial cells, surface mucus, epithelial layer, and immune defense. The epithelial cell layer and the mucin layer constitute the physical barrier; IAP and antibacterial proteins secreted by Paneth cells represent the functional barrier.^18^

IAP dephosphorylates substrates such as LPS and other pathogen-associated molecular patterns. Its role in reducing local intestinal inflammation and maintaining gut barrier function is reviewed later. The intestinal mucosal layer represents the first physical barrier. The small intestine harbors a single, tightly attached mucus layer, whereas in the colon, the mucus is composed of two layers: the essentially sterile inner layer which is firmly attached to the epithelial cells and the outer layer which contains commensal bacteria that prevent the entry of pathogenic bacteria. The epithelium is constituted of a single layer of different intestinal epithelial cells (IECs), and cells within the epithelial layer are sealed by tight junction proteins including claudins, zonula occludens-1 (ZO-1), and occluding, preventing paracellular transport.^19^ The tight junctions (TJs) constitute the major determinant of the intestinal physical barrier and can prevent the paracellular passage of large molecules through the epithelium.^20^

Disruption of the intestinal barrier can cause LPS translocation and cause endotoxemia in systemic circulation and chronic liver inflammation. The permeability of the epithelium is determined by the composition and abundance of different components of the TJs.^21^ Many factors can alter intestinal permeability, such as diet, alcohol intake, medication, and physiological factors such as age and stress.^22–24^ Changes in intestinal microbiota composition can influence intestinal permeability, as a study showing that a high-fat diet changed the gut microbiota content and could increase intestinal permeability. More importantly, this effect was completely restored by antibiotic treatment.^25^ And vice versa, probiotic bacteria and probiotic mixture are shown to have beneficial effects in reestablishing intestinal homeostasis and preserving epithelial barrier function.^26^ Bacterial metabolites, such as short-chain fatty acids (SCFAs), are also reported to play an important role in maintaining both intestinal immune functions and regulating gut barrier functions.^27^

LPS translocate the intestinal barrier mainly through the transcellular pathway, and chylomicrons can also bind and facilitate the absorption of LPS.^28^ Translocation of LPS can occur in physiological states.^29^ Under pathological conditions when there is an increase in gut barrier permeability, paracellular pathway of LPS is increased. The liver is the first organ in the body to encounter gut-derived bacteria and pathogen associated molecular patterns (PAMPs). Chronic exposure to increased levels of PAMPs has been linked to liver diseases.^30^

## LPS Conducts Liver Injury Through TLR4 Signaling Pathway

As the gut is considered to be the first barrier against bacteria, the liver is the second barrier based on the fact that the liver and the gut share both anatomical and functional relations and proximities; their close interaction is also described as the gut-liver axis.^31^ The portal venous system sits at the interface between the host and the inflammatory mediators that exist within the gut. The most important gut-derived inflammatory mediator, LPS, enters the liver through the portal vein and is detoxified in the liver. Only few LPS passes through the gut barrier and finally arrives in the liver under physiologic conditions. However, small intestinal bacteria overgrowth (SIBO) or an increase in intestinal permeability leads to translocation of bacteria and its by-products such as LPS. After arriving in the liver, LPS is taken up by hepatocytes and Kupffer cells and is excreted into the bile duct in further process.^32^ In patients with chronic liver disease, high LPS portal/peripheral concentrations have been observed.^33^ LPS binds to LPS-binding protein (LBP), and the LBP-LPS complex is transferred to membrane bound or soluble cluster of differentiation 14 (CD14), thereby specifically binds to toll-like receptor 4 (TLR4) and induces the interaction of TLR4 with adaptor molecule myeloid differentiation factor 88 (MyD88). MyD88 further activates downstream mitogen-activated protein kinase (MAPK) and nuclear factor-κB (NF-kB). Stimulation of the LPS-TLR4 signaling pathway eventually leads to the release of proinflammatory mediators like tumor necrosis factor-α (TNF-α) and interleukin 6 (IL-6).^34^ LPS also mediates signaling through MyD88-independent pathway, but the activation of MAPK and NF-kB occurs in a delayed manner.^35^ In both ALD and NAFLD progression, TLR4 signaling is considered a key pathway, and, very interestingly, it is reported that mice deficient in TLR4 are resistant to both alcohol-induced liver injury and NAFLD.^36,37^

TLR4 is expressed on all types of liver cells, including Kupffer cells, hepatocytes, hepatic stellate cells (HSCs), and also cholangiocytes. Its expression on these cells is correlated with activation of fibrogenic cells and the stage of fibrosis.^32^ In normal liver, hepatic cells express minimal TLRs; therefore, the liver has a high tolerance to TLR ligands. Hepatocytes directly clear LPS as data showed that fluorescence was revealed in hepatocytes 5 min after injection of fluorescein isothiocyanate (FITC)-LPS into the portal vein and then rapidly secreted into the bile.^38^ LPS promotes TNF-α production in Kupffer cells. The inflammatory mediator TNF-α is considered a central mediator in the pathogenesis of both ALD and NAFLD.^39^ Despite the fact that Kupffer cells are the main targets of LPS in the liver, it is the HSCs to promote TLR4-dependent fibrosis.^40^ LPS can activate HSCs in vitro and in vivo, and Kupffer cells strongly enhance this process by producing transforming growth factor beta (TGF-β) and increasing the sensitivity of HSCs to TGF-β. Recent studies have pointed out that platelets can also play a role in liver injury as biopsy results showed that the number of platelets is associated with disease severity in NAFLD.^33^ Platelets passing through hepatic sinusoids can be activated by LPS and the number of TLR4+ platelets is positively correlated with serum LPS level in NAFLD patients, suggesting that LPS may activate platelets through TLR4 signaling pathway. LPS is capable of activating platelets via TLR4-mediated over-production of eicosanoids, and mice given aspirin showed a decrease of liver fibrosis.^41,42^

## Histology of ALD and NAFLD

**Fig. 1:**
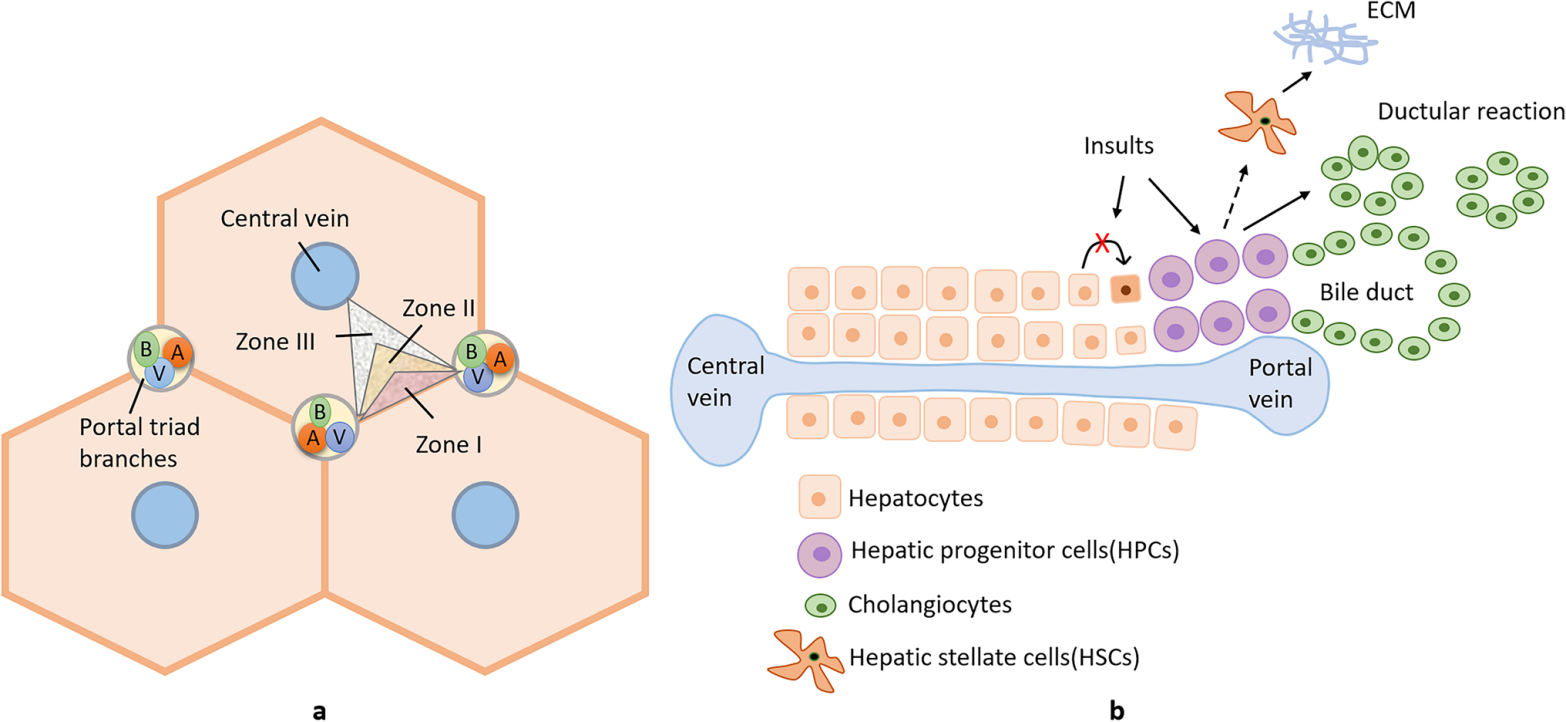
Histological zones of liver lobules (**A**) and the ductular reaction (**B**). **A** The liver can be divided functionally into three zones. Zone I is located around the portal triad, which is the most nutrient-oxygenated region. Zone III is located around the central vein, where oxygenation is poor. Zone II is located in between. **B** In chronic liver injury, hepatocyte regeneration is impaired and is replaced by the second pathway of HPCs activation. HPCs are bipotential cells and can differentiate into hepatocytes and cholangiocytes. The latter process causes the ductular reaction (DR). The HPCs can also interact with hepatic stellate cells, which are the primary source of the extracellular matrix (ECM) and the key players of the liver fibrogenic response

ALD shares histological similarities to NAFLD,^43^ indicating that there may be a common pathway for liver injury in both diseases. The hallmark of NAFLD and ALD is the excessive fat accumulation in the hepatocytes, which may be an isolated event (simple steatosis) or accompanied by evidence of inflammation and cell injury with or without fibrosis (NASH and ASH)^44^. The histological changes in ALD or NAFLD are lobulocentric (typically affecting zone 3) but not portal-based,^45^ including hepatocyte ballooning, Mallory bodies, zone 3 inflammation, and perisinusoidal fibrosis. Recent studies have identified the importance of portal fibrosis in predicting the subgroup of NAFLD patients that develop progressive liver disease and liver-related mortality.^46^ Portal inflammation is associated with portal-based changes, such as ductular reaction (DR), a reactive lesion at the portal tract interface comprising small biliary ductules with an accompanying complex of stroma and inflammatory cells.^47^ The underlying mechanism of DR is the activation of a secondary proliferative pathway of hepatic progenitor cells (HPCs) during the replacement of necrotic and apoptotic hepatocytes.^48^ Under regular conditions, the primary pathway of liver regeneration is maintained by the replication of adjacent hepatocytes within the lobules. With an insult of toxins, viral infection, alcohol, etc., the primary pathway is blocked and replaced by the secondary pathway: HPCs proliferate and differentiate into hepatocytes and bile ductal epithelia; the by-product is the DR (Figure 1).

Odena et al.^49^ proved that the LPS-TLR4 pathway stimulates the expansion of ductular reaction in alcoholic hepatitis (AH). The study found that the keratin 23 (KRT23) gene, which is expressed in the ductular reaction cells, is the most upregulated gene in AH compared to NASH and normal livers. They discovered that LPS administration markedly induced KRT23 expression in mice. This effect can be attenuated in TLR4-deficient mice, further indicating that the LPS-TLR4 pathway mediates the development of ductular reaction in chronic liver injury.

Another study by Vespasiani-Gentilucci et al.^32^ provided evidence that serum LPS levels correlated with portal/interface inflammation, the activity of portal/septal myofibroblasts, and fibrosis in NAFLD patients. Using immunohistochemistry, TLR4 expression was observed in hepatic progenitor cells, biliary cells, and portal/septal macrophages. TLR4-positive hepatic progenitor cells and bile ducts/ductules correlate with portal/interface inflammation, activation of fibrogenic cells, and fibrosis, implicating that the LPS-TLR4 pathway is associated with inflammation and fibrosis progression in NAFLD.

## LPS in the Pathogenesis of NAFLD

**Fig. 2:**
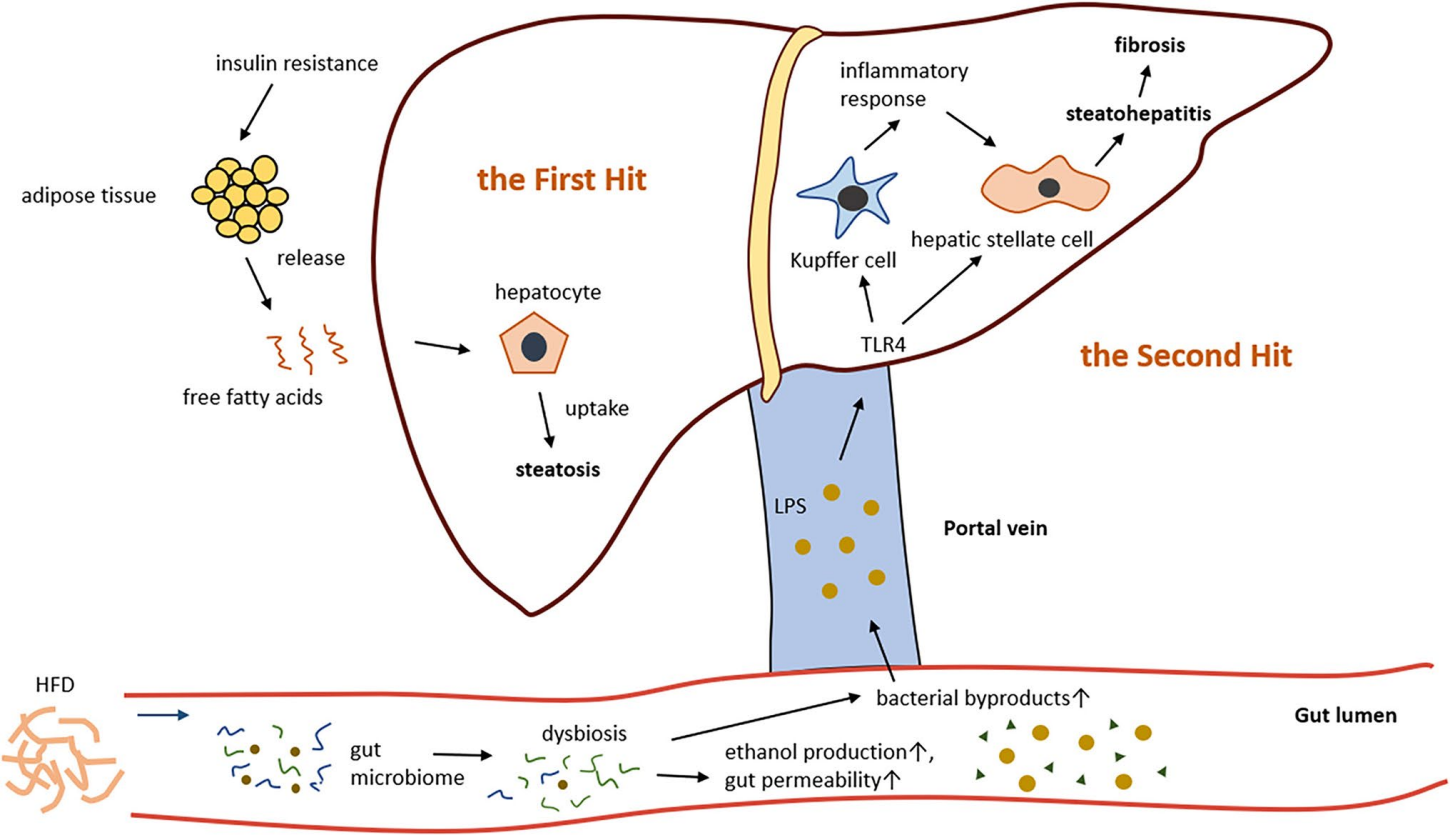
LPS acts as the second hit in the pathogenesis of NAFLD. Insulin resistance is the major factor for the progression of NAFLD, leading to an increase in free fatty acids (FFAs) in the circulating blood. Excessive uptake of FFAs by hepatocytes results in steatosis, making the liver more vulnerable to further insults, which is considered the “first hit.” A high-fat diet (HFD) could lead to gut dysbiosis, which further causes an increase in bacterial by-product production and increased gut permeability. Lipopolysaccharide (LPS) translocates the gut barrier, enters the liver through the portal vein, and activates Kupffer cells and hepatic stellate cells (HSCs) through the LPS-TLR4 pathway, resulting in an inflammatory response which leads to steatohepatitis and eventually fibrosis

NAFLD is the liver manifestation of metabolic syndrome and is characterized by massive ectopic triglyceride accumulation in the liver in the absence of any other liver disease or significant alcohol consumption.^50^ The etiology of NAFLD is previously referred to as a “two-hit” hypothesis:^51^ The first hit involves lipid accumulation in the hepatocytes, making the liver more vulnerable to toxins. Further insults like ethanol and LPS can act as the second hit and amplify the initial stress, causing oxidative stress and inflammatory response within the liver, resulting in the development of steatohepatitis (Figure 2). The “two-hit” hypothesis suggests that NASH is generally preceded by simple steatosis. But this view seems to be too simple to elucidate the complexity of the pathogenesis of NAFLD. Recently, it became widely accepted that the pathogenesis of NAFLD is a “multiple parallel hits” process.^52^ In this hypothesis, various hits (genetic and environmental factors) may occur parallelly, leading to liver inflammation. Among these hits, gut-derived factors appear to play a central role. Although traditional views consider that simple steatosis and NASH are consecutive changing processes, emerging evidence indicates that these two diseases can arise as independent conditions.^53,54^ Studies have found that the patients with simple steatosis will not always develop NASH and that the two diseases have a huge difference in prognosis in long term follow-up. This evidence further demonstrates that steatosis may not always precede inflammation.

Insulin resistance (IR) has been recognized as a major factor in the development of NAFLD.^55^ IR is thought to contribute to NAFLD by enhancing lipolysis of the adipose tissue, resulting in the increase of free fatty acids flux into the liver.^56^ High-fat, high cholesterol diets can result in endotoxemia and low-grade inflammation in both human and animal models.^57,58^ Lifestyle modifications such as diet, physical exercise, and weight loss are advocated. The Mediterranean diet characterized by reduced carbohydrate intake and increased monounsaturated and omega-3 fatty acid intake has a beneficial effect in overweight patients with NAFLD.^59^ A previous study showed that even short-term diet modification could reduce liver steatosis and steatohepatitis.^60^

Endotoxin plasma level is significantly higher in NAFLD patients, and it seems that endotoxin level is associated with the severity of hepatic steatosis.^61^ Gut dysbiosis is causative for the enhanced secretion of LPS and the resulting inflammation in NAFLD development.^62^ Generally, dysbiosis is defined as an imbalance or alteration in the microbiota that can have an unfavorable effect on the host.^63^ A previous study investigated taxonomic compositions of gut microbiota within the spectrum of NALFD lesions and found that more serious NAFLD lesions (NASH and significant fibrosis) associate with gut dysbiosis.^64^ The study also found that increased levels of *Bacteroides* were independently associated with NASH and increased *Ruminococcus* abundance with fibrosis, linking these two taxa of bacteria to the severity of NAFLD.

The gut microbiota composition of an individual is a kind of a fingerprint highly influenced by the type of diet.^65^ It has been shown that microbiota play an important role in nutrition intake and patients with obesity or metabolic disorder appear to have an intestinal microbiota signature that can harvest more energy than their healthy counterparts.^66^ Changes in abundance and diversity of the gut microbiota in NAFLD have also been characterized. Though many studies have reported conflicting results of the microbiome signatures in NAFLD, an increase in *Firmicutes* and a reduction in *Bacteroidetes* is found in most studies.^67^ Thus, a higher Firmicutes/Bacteroidetes ratio is frequently cited as a hallmark of obesity.^68^ Furthermore, the amount of ethanol-producing bacteria (e.g., *Escherichia coli*) was reported to be increased in NAFLD, which may cause increased intestinal permeability and increased translocation of endotoxins from the intestinal lumen to the portal blood.^69^ The impact of microbiota on liver disease is further supported by experiments during which bacterial transfer from the human gut to germ-free (GF) mice resulted in metabolic diseases^70,71^

Guo and collegues^72^ found that LPS per se can cause an increase in intestinal epithelial tight junction permeability in vitro and in vivo. This effect was mediated by an increase in enterocyte TLR4 expression and a TLR4-dependent increase in membrane CD14 expression. Studies have shown that physiologically relevant concentrations of LPS (0 to 2000 pg/mL) cause an increase in intestinal epithelial TJ permeability.^73,74^ These findings indicate that LPS is an important pathogenic factor in the intestinal inflammatory process.

The gut microbiota is influenced by various factors, including the genetic background of the host, type of diet, age, and medication. How these factors exactly affect gut microbiota composition and function is not fully understood with several studies presenting contradictory data. The regulation of gut microbiota provides a new insight into the treatment of NAFLD.

## LPS in the Pathogenesis of ALD

ALD is associated with high morbidity and mortality rates. Most chronic heavy drinkers develop steatosis, but only 35% develop advanced liver disease.^75^ A previous study showed that intestinal hyperpermeability occurs only in alcoholics with ALD and not in those without liver disease.^76^ Furthermore, chronic alcohol administration has been shown to increase gut-derived endotoxin levels in the portal circulation.^77^ This indicates that alcohol plays a crucial role in promoting intestinal hyperpermeability resulting in endotoxemia, systemic inflammation, and liver damage. Alcohol consumption destroys the integrity of the intestinal barrier, disturbs the gut microbiota, and is associated with an increase in the abundance of endotoxin-producing bacterial types.^78^ Metagenomic analysis of the intestinal microbiome of individuals with a history of chronic alcohol abuse has revealed reduced bacterial diversity and a lower proportion of *Bacteroidaceae* and probiotic bacteria such as *Lactobacillus* ^79,80^. Patients with alcoholic cirrhosis have an increased relative abundance of *Enterobacteriaceae*.^81^ Alcohol consumption affects bacterial composition within specific phyla. A study by Llopis et al.^82^ found that mice harboring the intestinal microbiota from patients with severe alcoholic hepatitis developed more severe liver inflammation, indicating that individual susceptibility to ALD is substantially driven by the intestinal microbiome.

Both chronic alcohol consumption and acute alcohol intake (binge drinking) can impair the intestinal barrier and increase serum levels of bacterial products.^76,83^ Endotoxemia in ALD was first recognized by the detection of antibodies against *Escherichia coli* in the plasma of patients with ALD.^84^ Humans and animals with chronic alcohol consumption develop a “leaky gut,” as evidenced by higher levels of plasma endotoxin. The plasma endotoxin levels are also associated with the severity of disease.^85^ Alcohol damages specific components of the intestinal barrier such as proteins involved in innate antibacterial defense. Animal experiments have shown that chronic exposure to ethanol can cause gut-barrier disruption featured by a decrease in tight junction protein ZO-1 and occludin expression.^86,87^

Besides ethanol, the by-product acetaldehyde from the metabolization of alcohol by gut microbiota appears to play a crucial role in gut barrier dysfunction. At concentrations ranging from 99 to 760μM, acetaldehyde increases the paracellular permeability of Caco-2 cell monolayer.^88^ The underlying mechanism includes the dissociation of ZO-1 from the junctions in acetaldehyde-treated cell monolayer, thus leading to disrupted TJ structure and the increase in paracellular permeability.^89^ The study further revealed that acetaldehyde increases tyrosine phosphorylation of ZO-1, E-cadherin, and β-catenin, resulting in disruption of the TJs.

Chronic alcohol consumption is also associated with changes in bile acid profiles. ALD patients have an increased secondary bile acid formation.^90^ A study by Xie et al.^91^ showed that ethanol consumption could lead to a substantial decrease in taurine-conjugated (hydrophilic and less toxic) bile acids, resulting in impaired lipid emulsification and liver steatosis in mice.

## Intestinal Targeted Therapy

While simple steatosis is considered a “benign disease,” treatment of NAFLD and ALD should focus on NASH and ASH. Currently, there is no targeted and thus effective drug therapy for the treatment of NASH or ASH. Especially, the long-term intake of traditional treatments for chronic liver diseases such as antibiotics and corticosteroids is associated with various side effects for patients. There is accumulating evidence that the interplay between the gut microbiota and the liver is critical in the pathogenesis of chronic liver disease. Hence, there is a large interest in modulating the microbial community to achieve a therapeutic effect or even reverse liver fibrosis. The manipulation of the gut microbiome by fecal microbiota transplantation (FMT), probiotics, prebiotics, and synbiotics was shown to have a beneficial effect in improving liver phenotype in patients with chronic liver disease. IAP as a naturally occurring brush boarder enzyme that detoxifies LPS and preserves microbial homeostasis and gut barrier integrity appears to be a promising candidate for treatment of liver fibrosis in patients with NAFLD or ALD.

## Fecal Microbiota Transplantation

This procedure involves the transfer of processed feces to restore a “healthy microbiome.” FMT has been successfully used to treat recurrent *Clostridium difficile* infection (rCDI) for years^92,93^ and possesses potential in treating gastrointestinal as well as extraintestinal diseases.

FMT has shown an effect in alleviating high-fat diet-induced steatohepatitis in mice.^94^ The therapeutic effect may be caused by an increase in “beneficial” gut microbiota, improving the tight junction of small intestinal and lowering the LPS levels. Ferrere et al.^95^ performed FMT from alcohol-resistant donor mice (alcohol-fed mice did not develop alcohol-induced liver lesions) to alcohol-sensitive receiver mice (alcohol-fed mice developed liver lesions) and found that FMT protected the alcohol-sensitive mice from alcohol-induced depletion of *Bacteroides*. FMT treatment was also found to prevent steatosis in alcohol-fed mice, indicating the beneficial effect of FMT in preventing ALD development. In a small-scale pilot study conducted by Philips et al.,^96^ 8 patients with steroid-ineligible severe alcoholic hepatitis received FMT treatment from healthy donors. The results showed that indices of liver disease severity, including ascites, hepatic encephalopathy, and mean bilirubin, significantly improved after FMT. FMT treatment also showed an effect on modulation of gut microbiota and improved prognosis in these patients. In a recent randomized trial, twenty patients with liver cirrhosis were randomly given FMT capsules or placebo. Patients receiving FMT capsules showed an improved gut microbial function and significantly reduced systemic inflammation markers (IL-6 and LBP).^97^

FMT contributes to restore a balanced gut microbiota composition and has emerged as a therapeutic option for chronic liver disease. Although FMT shows its beneficial effect in animal models and clinical trials of non-alcoholic and alcoholic liver steatosis, it has not been approved for clinical use. Further research is needed to verify the safety and effectiveness of FMT in chronic liver disease.

## Probiotics

Probiotics are defined by the Food and Agriculture Organization (FAO)/World Health Organization (WHO) as living microorganisms that confer a health benefit on the host when administered in adequate amounts.^98^ They exhibit characteristics including tolerance to gastrointestinal conditions, ability to adhere to the gastrointestinal mucosa and competitive exclusion of pathogens.^99^ The strains most frequently used as probiotic bacteria belong to the *Bifidobacterium* and *Lactobacillus* genera and are widely used in yogurts and other dairy products.^100^ Probiotics are generally considered to have a beneficial effect on the human intestine. Induction of probiotics may control the growth of pathologic organisms. Studies have proven that probiotics are efficient in treating various diseases such as antibiotic-associated diarrhea (ADD), inflammatory bowel diseases (IBD), and neuropsychiatric disorders. ^101–103^

Probiotics have been proven to lower the plasma triglyceride (TG) levels in animal models of metabolic syndrome.^104^ Plaza-Diaz et al.^105^ demonstrated the reduction in liver steatosis of obese mice fed certain probiotic strains. This effect was associated with lower serum LPS levels as matched trends were observed in LPS serum concentration and liver TG content.

Clinical trials have confirmed the beneficial effects of probiotics in nonalcoholic liver diseases both in adults and children.^106–108^ Probiotic treatment has been shown to play a role in improving liver function. The intake of probiotics leads to reduced levels of alanine aminotransferase (ALT), aspartate aminotransferase (AST), glutamine transferase (GGT), total cholesterol (TC), TG, and improved liver histological marker such as NAS (NAFLD Activity Score).^107^ Probiotic supplementation is also able to improve biomarkers of inflammation (e.g., TNF-α, IL-6) and steatosis (e.g., arginase, prolidase).^109^

Similar to NAFLD, patients with alcoholic liver disease can also benefit from a probiotic treatment. In a Russian pilot study^110^, *Bifidobacterium bifidum* and *Lactobacillus plantarum 8PA3* were randomly given to patients diagnosed with alcoholic psychosis. The treatment led to the alterations in bowel flora in alcoholic patients and liver enzyme reduction, indicating that probiotics can improve alcohol-induced liver injury. Other randomized-controlled trials of *Lactobacillus casei* supplements in patients with alcoholic liver injury support this finding.^111^

In general, the mechanisms by which probiotics prevent chronic liver injury include: reducing gut-derived microbial LPS by restoring the bowel flora, repairing the intestinal mucosa and barrier function, modulation of the immune system, and reducing inflammatory cytokine levels.^112^

## Prebiotics and Synbiotics

A prebiotic is a non-viable food component that can confer a health benefit on the host that is based on the modulation of the intestinal microbiota.^113^ Main prebiotics include primarily short- and long-chain fructans (fructo-oligosaccharides (FOS) and inulin), galacto-oligosaccharides (GOS), and lactulose.^114^ These substances possess features including non-digestibility, fermentation by intestinal microflora, and selective stimulation of growth and activity of intestinal bacteria.^115^

Prebiotics have beneficial effects on the gastrointestinal tract, which include prevention of pathogen damage or immune system modulation, improvement of gut barrier function, reduction in the pathogenic bacteria population, and the production of SCFAs.^116^ Prebiotics are used mostly as a selective medium for the growth of a probiotic strain, and *Lactobacilli* and *Bifidobacteria* are the usual target genera for prebiotics. Large bundles of studies have shown that prebiotics are able to increase the composition and/or activity of *Lactobacillus* and *Bifidobacterium* populations.^117–120^ In lipid metabolism, prebiotics exhibit serum or hepatic lipid-lowering properties, and are now considered a potential dietary adjunct in reducing the risks of cardiovascular diseases (CVD) with minimal side effects^121^. A randomized placebo-controlled trial carried out in Canada showed that, after 16 weeks of consumption of oligofructose-enriched inulin, there was a significant decrease in body weight *z*-score, percent body fat, and percent trunk fat and serum level of IL-6 in healthy children with overweight or obesity compared to the placebo control group.^122^ The study also observed a significantly higher abundance of *Bifidobacterium* spp. in the prebiotic consumption group, indicating that prebiotic consumption could selectively alter gut microbiota.

The prebiotic inulin is proven to be effective in preventing NAFLD in animal experiments.^123,124^ However, the effect of inulin in human trials remains controversial. Chambers et al.^125^ explored the effects of dietary supplementation with inulin in adults with NAFLD and found that inulin consumed at 20 g/d increased intrahepatocellular lipid (IHCL). They speculated that the acetate derived from colonic fermentation of inulin could provide an additional lipogenic substrate to the liver.

A synbiotic is a mixture of 1 or more probiotics and 1 or more prebiotics that beneficially affect the host by promoting the survival and colonization of the live microbes in the gastrointestinal tract.^115^ It is known that probiotics are active in the small and large intestine and the effect of a prebiotic is mainly in the colon. The combination of the two may have a synergistic effect.^126^ The intake of synbiotics has been demonstrated to modify the composition of the microbiota, thus protecting against inflammation and hepatocyte damage.^127^ A recent meta-analysis involving 15 randomized clinical trials in which 8 studies about synbiotics were included showed that synbiotics supplementation can improve TC, TG, high-density lipoprotein (HDL), and low-density lipoprotein (LDL) in patients with NAFLD, indicating that synbiotics could improve lipid profiles in those patients.^128^ Among these studies, Malaguarnera et al.^127^ conducted a randomized clinical trial in which 66 NASH patients were enrolled and randomly given *Bifidobacterium longum* with fructo-oligosaccharides or placebo. The results showed that synbiotic treatment improved liver histology in NASH patients and had a beneficial effect in reducing inflammation markers like C-reactive protein (CRP) and TNF-α. These effects may be due to reduced LPS exposure to the liver.

Though studies of the relationship between synbiotics and ALD are much lesser, the beneficial effect of synbiotics in attenuating chronic alcohol intake induced liver injury is observed in mice.^129,130^ Synbiotic supplementation was also shown to decrease serum LPS levels in high-risk alcoholic participants and in patients with alcohol-related cirrhosis.^131,132^

## Intestinal Alkaline Phosphatase

IAP is ubiquitously expressed by enterocytes in the proximal small intestine and exists in high concentrations within luminal vesicles secreted by enterocytes on the brush border of the microvilli.^133^ IAP is secreted bilaterally and is also released in small amounts into the blood.^134^ IAP plays an important anti-inflammatory role by dephosphorylating potentially pro-inflammatory ligands such as adenosine triphosphate (ATP), uridine diphosphate (UDP), unmethylated cytosine-guanosine dinucleotides (CpG), and LPS.^135^ Luminal IAP can prevent and reduce intestinal inflammation and bacterial translocation and is considered the first layer of the intestinal barrier.^18^ The anti-inflammatory effect makes it a potential treatment for various diseases. Previous studies had found decreased levels of IAP protein expression in inflamed colonic mucosa in children with IBD and decreased IAP mRNA levels in inflamed tissue of adults with IBD.^136,137^ Exogenous IAP supplementation showed a beneficial effect in alleviating inflammation in a rat model of IBD.^138^ In a phase II trial, intravenous injection of IAP improved renal function in patients with severe sepsis and septic shock.^139^ And another phase II trial demonstrated that administration of bovine IAP could reduce post-surgical inflammatory response in patients undergoing coronary artery bypass grafting (CABG).^140^

**Fig. 3:**
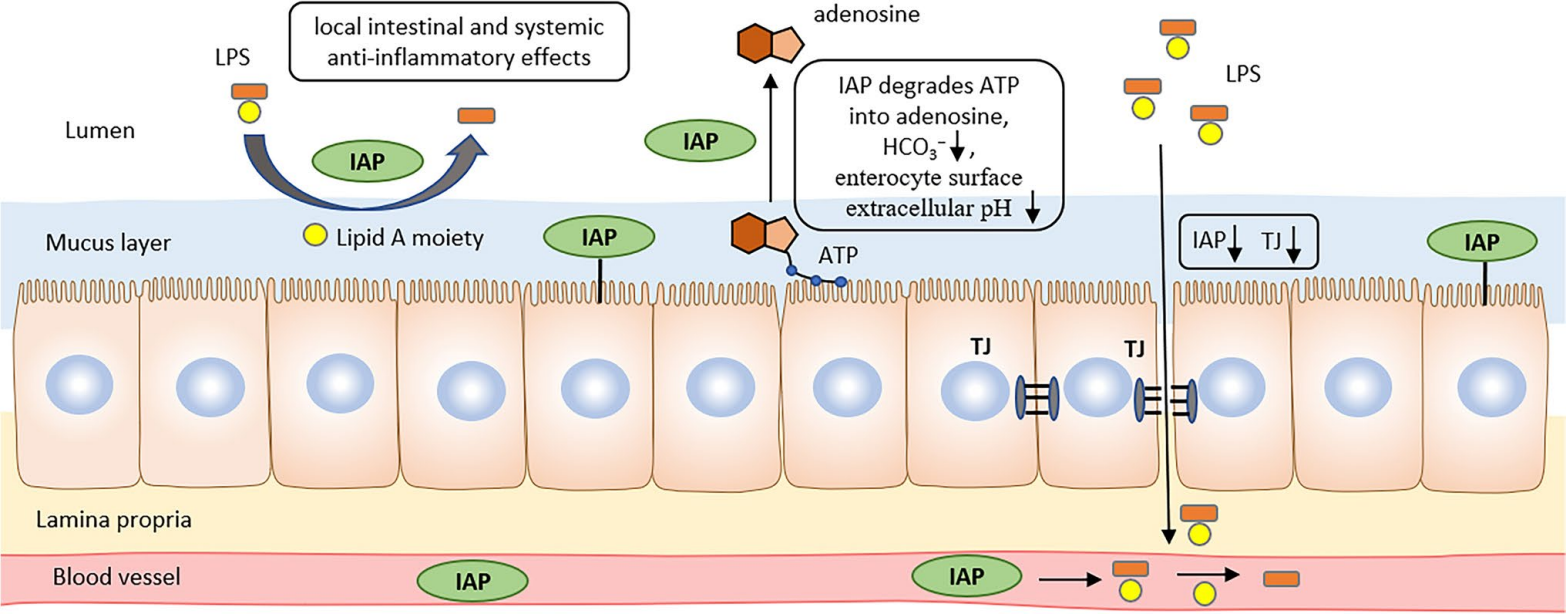
The role of IAP in preventing local inflammation, preventing LPS translocation, and regulation of enterocyte surface extracellular pH. IAP is highly expressed in the brush border membrane of duodenal epithelial cells and is secreted bilaterally into the gut lumen and the blood. IAP can detoxify LPS, resulting in amelioration of intestinal and systemic inflammation. ATP serves as a substrate for brush border IAP. In the gut lumen, the presence of ATP increases HCO3—secretion. IAP decreases luminal ATP concentration and diminish this pathway. IAP also plays a role in regulating tight junction protein levels, preserving gut integrity and preventing translocation of bacterial by-products

IAP is an important enzyme to maintain the integrity of the gut barrier. By dephosphorylating luminal ATP, IAP can act as a component of the ecto-purinergic signaling system to regulate ATP-dependent HCO_3_^−^ secretion and localized extracellular pH (pH_0_).^141^ Liu et al.^142^ demonstrated that IAP can directly regulate TJ protein levels as its gene depletion in mouse embryonic fibroblasts resulted in significantly lower levels of ZO-1, ZO-2, and occludin expression, and IAP overexpression in Caco-2 and T84 cells resulted in increases in mRNA levels of ZO-1 and ZO-2 (Figure 3). Another study showed a similar result that the intestinal tissue of IAP-KO mice presents a significant decrease in TJ proteins; moreover, oral supplementation with IAP restores TJ protein expression.^143^

IAP also plays a crucial role in regulating the gut microbiota. Malo et al.^144^ examined the status of the gut microbiota in IAP knockout (IAP-KO) mice and found dramatically fewer microbes in their stools compared with wild-type (WT) mice when fed a high-fat diet. Moreover, oral supplementation of calf IAP (cIAP) promoted the restoration of the normal gut microbiota following antibiotic treatment. Kühn et al.^145^ observed the beneficial effect of IAP supplementation in preserving the homeostasis of gut microbiota during aging in mice. IAP has been shown to prevent chronic liver disease in 2 different mice models. Liu et al. ^146^ demonstrated that fecal IAP activity decreases in humans with liver cirrhosis and oral supplement of IAP attenuated liver fibrosis in mice. Hamarneh et al.^147^ demonstrated that pretreatment with IAP attenuated the development of alcohol-induced fatty liver, decreased hepatic pro-inflammatory cytokines, as well as serum LPS levels, and prevented alcohol-induced gut barrier dysfunction in mice, indicating that oral IAP supplementation could present a novel therapy to prevent alcoholic-related liver disease. Though the beneficial effect of IAP in chronic liver diseases and maintaining gut barrier function has been observed in various preclinical studies, the therapeutic value of IAP in patients with chronic liver disease is not known.

## Conclusion

Accumulating evidence has pointed out the importance of the gut-liver axis in the development of liver disease. Translocation of bacterial by-products such as LPS results in chronic inflammation in the liver. Treatments targeting the gut microbiome such as FMT, probiotics, prebiotics, and synbiotics have shown an effect in attenuating liver inflammation. IAP possessing an anti-inflammatory effect by dephosphorylating LPS and maintaining gut barrier function is also showing therapeutic potential. Current evidence demonstrates that maintaining gut barrier integrity and microbiome homeostasis is of great significance in treating liver diseases.
